# An innovative technique for electronic transport model of group-III nitrides

**DOI:** 10.1038/s41598-020-75588-3

**Published:** 2020-10-30

**Authors:** Anshika Srivastava, Anshu Saxena, Praveen K. Saxena, F. K. Gupta, Priyanka Shakya, Pankaj Srivastava, Manish Dixit, S. Gambhir, R. K. Shukla, A. Srivastava

**Affiliations:** 1 Tech Next Lab Pvt Ltd, Lucknow, 226003 India; 2 Sanjay Gandhi Post Graduate Institute of Medical Sciences (Deemed Univeristy), Lucknow, 226014 India; 3 Department of Physics, University of Lucknow, Lucknow, 226007 India

**Keywords:** Materials science, Physics, Software

## Abstract

An optimized empirical pseudopotential method (EPM) in conjunction with virtual crystal approximation (VCA) and the compositional disorder effect is used for simulation to extract the electronic material parameters of wurtzite nitride alloys to ensure excellent agreement with the experiments. The proposed direct bandgap results of group-III nitride alloys are also compared with the different density functional theories (DFT) based theoretical results. The model developed in current work, significantly improves the accuracy of calculated band gaps as compared to the ab-initio method based results. The physics of carrier transport in binary and ternary nitride materials is investigated with the help of in-house developed Monte Carlo algorithms for solution of Boltzmann transport equation (BTE) including nonlinear scattering mechanisms. Carrier–carrier scattering mechanisms defined through Coulomb-, piezoelectric-, ionized impurity-, surface roughness-scattering with acoustic and intervalley scatterings, all have been given due consideration in present model. The direct and indirect energy bandgap results have been calibrated with the experimental data and use of symmetric and asymmetric form factors associated with respective materials. The electron mobility results of each binary nitride material have been compared and contrasted with experimental results under appropriate conditions and good agreement has been found between simulated and experimental results.

## Introduction

The group-III nitride semiconductor materials possess proven outstanding properties for device applications^1^. The high electric breakdown voltage, thermodynamic stability, high saturation current, strong piezoelectric polarization effect make them suitable for production of light emitting diodes (LED), laser diodes^1^ and high power transistors (HEMT)^2^. Solid state lighting sources based on nitride materials have shown tremendous potential in terms of less energy consumption (upto 85% less). The ternary nitride alloys provide flexibility to enhance the device performance through tailoring the material properties by changing material contents^1–6^.

The non-availability of sufficient quality materials impeded the technological advancement for group-III nitride based devices^6^. The transport properties at low and high-field conditions and the corresponding potential for device applications are yet to be uncovered^6^. Computational techniques appear to be most valuable tools for better understanding of the underlying physics and transport phenomenon under non-equilibrium conditions.

The present state-of-the-art in studying electronic transport in semiconductors have evolved to the use of band structure calculated using density functional theory (DFT), carrier–phonon interactions using similar ab-initio methods, carrier-impurity scattering using sophisticated methods to account for dielectric screening^3^. However, the computational tool based on DFT suffers with limitations that it can be used for a very small structure of clusters of atoms. The other big limitations of DFT are that many times it doesn't correctly treat the exchange interaction and long-range non-covalent interactions^4–8^. Semi-empirical methods appear to be most suitable technique to address these issues. Furthermore, it can be easily extended to simulate dynamical properties of the materials^4–11^.

Ensemble Monte Carlo (EMC) technique is the best suited numerical approach for accurate solution of the Boltzmann equation (BTE) under nonlinear response conditions^12–14^. The aim of the present paper is to demonstrate a simple and inexpensive theoretical approach for prediction of the low and high field mobility model for binary and ternary wurtzite nitride alloys for entire concentration range. The full electronic band structure is simulated by EPM method (using TNL’s FullBand simulator) for better understanding of the structural and electronic properties^15^. TNL’s FullBand Simulator is powerful tool to simulate full electronic band structures of semiconductors with the zincblende as well as wurtzite structures^15^.

The carrier transport properties including multiple appropriate scattering mechanisms^12–16^ associated with nitride alloys have been simulated by the solution of BTE through EMC method (using TNL’s ElecMob simulator)^12–17^. ElecMob simulator is capable to simulate carriers transport on full energy band including random scattering events due to impurities, lattice vibrations, etc.^17^. The difference in present analysis from earlier reported results is attributed to using optimized band structures and estimation of electron mobility based on different scattering mechanisms. Various scattering processes included in present analysis are—acoustic, intervalley, optical (Polar), piezoelectric, ionized impurity, Coulomb, surface roughness^16^.

The calculations of electronic band structure parameters are important prerequisite for the Monte Carlo method used here. We are presenting solution to address successfully the carrier transport in III–V wurtzite alloys using the Tech Next Lab’s, TNL’s ElecMob simulator^17^. For this the full electronic band structure has been calculated, using TNL’s FullBand simulator, from which different physical parameters have been determined. The electronic and optical properties extracted from full band structure are used as input parameters for obtaining solution of BTE which is required for simulation of carrier transport under external applied forces on the three valleys ${\Gamma }_{\mathrm{c}}^{1},\text{ U and }{\Gamma }_{\mathrm{c}}^{3}$. The proposed mobility model is calibrated against the reported experimental/theoretical results along with calibration of selected electronic structure parameters.

## Numerical technique

Empirical pseudopotential method (EPM) is able to provide behavior of electrons on full electronic band structures of the crystals with spin–orbit interactions and also provides flexibility to fit experimental transport data. The reliable description of the density of states (DOS) makes this method superior approach over the k.p and tight binding methods^18^.

The effective single-particle Schrödinger equation is^13^,

1 $$- \frac{{\hbar^{2} }}{{2{\text{m}}^{*} }}\vec{\nabla }^{2}\psi \left( {\text{r}} \right) + \left[ {{\text{V}}^{{\mathrm{c}}} \left( {\text{r}} \right) + {\text{V}}^{{\mathrm{e}}} \left( {\text{r}} \right)} \right] = {\text{E}}\psi \left( {\text{r}} \right)$$

Here V^c^(r) and V^e^(r) demonstrate about the intrinsic crystal potential, and the extrinsic potential respectively. In proposed model, the crystal potential is chosen by local atomistic empirical pseudopotentials of each atom α^13^,

2 $${\text{V}}^{{\text{c}}} \left( {\mathbf{r}} \right) = \mathop \sum \limits_{\alpha } {\text{V}}^{\alpha } \left( {\left| {{\mathbf{r}} - {\mathbf{R}}_{\alpha } } \right|} \right)$$

Here V_α_ is the radial empirical pseudopotential of αth atom centered at **R**_α_ as taken in reference^13^.

The electronic band structures of the binary wurtzite compounds are simulated using lattice parameters obtained from XRD studies as depicted in Table 1. The lattice constants of ternary alloys are computed through interpolation of lattice constants of binary alloys with inclusion of virtual crystal approximation (VCA) techniques^9–11^. The selected band parameters are matched with the reported band parameters for different mole fraction of ternary alloys. The energy band gap values at gamma valley, simulated using only interpolated lattice constants ‘a’ of associated binary alloys for ternary are shown in Table 2. Different researchers have taken different values of bowing parameters for calibration of band gap parameter^19–22^. It is important to mention here that previous results have been reported using interpolation of energy band gap of binary nitride alloys using bowing parameter (used as fitting parameter^19–22^) whereas in the present work interpolation of lattice constant of binary nitride alloys has been used along with alloy-disorder effect under VCA, to calculate the accurate lattice parameter of ternary alloy^19–22^.

**Table 1:** Lattice constants and internal parameters of binary group-III nitrides used in the present analysis.

Material	a (Å)	c (Å)	u
GaN	3.189^10^	5.185^10^	0.377^10^
AlN	3.110^10^	4.980^10^	0.380^10^
InN	3.544^10^	5.718^10^	0.379^10^

**Table 2:** Optimum alloy disorder parameters used in current study and interpolated lattice constant of ternary nitrides for x = 0.2.

Material	Al_0.2_Ga_0.8_N	In_0.2_Ga_0.8_N	In_0.2_Al_0.8_N
Alloy disorder (P)	0.025^32^	0.022^32^	0.025^32^
Simulated Lattice Constant (Å)	3.169	3.256	3.193

The lattice constant has been simulated using the formula^9^ $\mathrm{a}\left(\mathrm{ABN}\right)=\mathrm{x}\cdot \mathrm{a}\left(\mathrm{AN}\right)+\left(1-\mathrm{x}\right)\cdot \mathrm{a}\left(\mathrm{BN}\right)+\mathrm{P}\cdot {[\mathrm{x}\cdot \left(1-\mathrm{x}\right)]}^{1/2}$.

**Figure 1:**
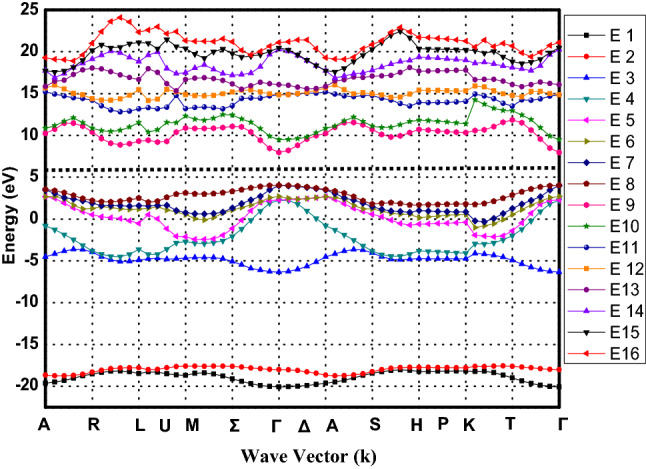
Full electronic band structure of Al_0.2_Ga_0.8_N at 300 K simulated by empirical pseudopotential method using virtual crystal approximation.

For brevity, we have depicted only Al_0.2_Ga_0.8_N full band structure here in Fig. 1. To better validate the accuracy of proposed empirical pseudopotential method inbuilt in FullBand simulator^15^, used for the full band structure simulation in current manuscript, the authors have compared and contrasted the energy values ${\Gamma }_{8}^{\mathrm{v}},\mathrm{ and }{\Gamma }_{1}^{\mathrm{c}}$ at gamma valley with previously reported experimental data taken from references^5,7,23–27^. Band gaps for AlN, GaN, InN, and Al_0.2_Ga_0.8_N, In_0.2_Al_0.8_N and In_0.2_Ga_0.8_N alloys are also compared with the results obtained by various density functional theories including LDA^5,7,28^, LDA-1/2^5^, PBE^7,12,28^ and HSE^7,12,28^ methods. All the comparison data are tabulated in Table 3. The comparison reflects that our bandgap results for binary and ternary group-III nitrides are much closer to the experimental results and superior to the bandgap results obtained on basis of various DFT based approaches.

**Table 3:** Energy difference ${E}_{g}=({\Gamma }_{8}^{\mathrm{v}}-{\Gamma }_{1}^{\mathrm{c}})$ obtained using FullBand simulator based on the proposed model compared with those reported earlier, with those computed from previously reported various density functional theory (DFT) techniques and with experimental results.

Material	Previously reported values of E_g_	E^LDA^	E^LDA-1/2^	DFT^PBE^	DFT^HSE^	Experiments	E^This work^
AlN	6.54^7^, 6.23^10^	4.50^5,7^	6.06^5^	4.13^7^, 4.02^20^	6.42^7^, 6.29^20^	6.23^5^, 6.026^17^, 6.1–6.2^7,20^	6.20
GaN	3.5^7^, 3.507^10^	2.02^5^, 2.11^7^	3.52^5^	1.69^7,20^	3.55^7^, 3.55^20^	3.507^5^, 3.35^22^, 3.51^20^	3.47
InN	0.7–1.0^7,8^, 0.7–1.9^10^	− 0.03^5^, − 0.24^7^	0.95^5^	− 0.42^7,20^	0.86^7^, 0.86^20^	0.7–1.9^5^, 0.6–0.7^20^	0.7
Al_0.2_Ga_0.8_N	3.99*	2.353^5^	3.951^5^	4.570^12^	4.569^12^	3.962^24^	3.94
In_0.2_Ga_0.8_N	2.72–2.78*	1.52^5^	2.76^5^	2.272^5^	1.925^12^	2.625^23^	2.66
In_0.2_Al_0.8_N	4.7–4.76*	3.431^5^	4.409^5^	3.445^12^	2.976^12^	4.515^25^	4.71

Here suffix represents the reference numbers.

*LDA* local density approximation, *LDA-1/2* approximately includes the self-energy of excitations in semiconductors, *PBE* Perdew–Burke–Ernzerhof (PBE) exchange energy theory, *HSE* Heyd–Scuseria–Ernzerhof exchange–correlation functional uses an error function screened Coulomb potential to calculate the exchange portion of the energy.

*calculated from modified Vegard’s law^11,12^ ${\mathrm{E}}_{\mathrm{ g}}\left(\mathrm{x}\right)=\mathrm{x}\cdot {\mathrm{E}}_{\mathrm{g}}^{\mathrm{A}}+\left(1-\mathrm{x}\right)\cdot {\mathrm{E}}_{\mathrm{g}}^{\mathrm{B}}-\mathrm{b}.\mathrm{x}.(1-\mathrm{x})$ where A and B represent band gap values for binary nitride alloys and b is bowing parameter^12^.

The carrier transport on three valleys in ElecMob simulator are described by solution of Boltzmann transport equation (BTE) under non-equilibrium conditions due to applied external forces including rate change of distribution function. The Monte Carlo program implemented in ElecMob simulator for simulation of carrier transport process is initiated with carriers under equilibrium conditions, the first free flight duration is chosen with a probability distribution determined by the scattering probabilities under external electrostatic force. The force on each particle in real space is related to the E–k dispersion relation obtained with full band structure. Contribution of magnetic field B is taken as zero i.e. B = 0 in the present model^29,30^.

3 $$v = \frac{1}{\hbar }\frac{dE}{{dk}}$$

4 $${\text{k}}\left( {\text{t}} \right) = {\text{k}}\left( 0 \right) - \frac{{{\text{e}}\left(\varepsilon \right){\text{t}}}}{\hbar }$$

Here k is the time dependent wave vector, ħ is Planck’s constant, e and $\varepsilon$ are the electronic charge and external applied field respectively.

The electron–lattice, electron–electron and electron–defect coupling strengths are important physical quantities dictating the interactions of the electron with lattice and with extrinsic defect. These interactions are responsible for relaxing momentum and the energy of the electrons, and are included in terms of different scattering probabilities in the simulation program. Scattering rates are strongly dependent on the electronic structure. Density of states in particular valley at particular time plays important role in deciding the probability of scattering events which follow the energy conservation principle i.e. Fermi Golden rule.

All physical quantities of interest, e.g. velocity, energy associated with electron are recorded for the free flight of carriers. The free flight of carriers is interrupted by any one of the scattering processes and scattered electron goes into new k state which is randomly chosen as initial state for next free flight under repeated iterative scheme. It is found that the accuracy of calculated results depends on the precision in time scale for iteration.

## Results and discussion

This section concentrates on the results obtained regarding specific aspects of electron mobility investigated using the model proposed in this paper, which is based on wide band gap nitride alloys, under various operating conditions. Full band structure of Al_0.2_Ga_0.8_N has been shown here in Fig. 1. At normal pressure in wurtzite structure four atoms exist in the unit cell which produces eight valence bands. The projected density of states (per unit energy) and carrier effective masses at different valleys have been extracted from full band structure (not shown here) using energy-wavevector (E–k) data. In Al_0.2_Ga_0.8_N full band structure, bands 1 and 2 show strong s-like character and rest of the bands exhibit p-like character, Fig. 1. Especially bands 6 and 8 illustrate pure pxy-like and pz-like characters respectively. The primary valley for all materials lies at ${\Gamma }_{\mathrm{c}}^{1}$. The secondary valleys included in the simulation are located at U and ${\Gamma }_{\mathrm{c}}^{3}$. It should be noted that their relative energy ordering varies among the materials studied here—GaN, AlN, InN, Al_0.2_Ga_0.8_N, In_0.2_Ga_0.8_N and In_0.2_Al_0.8_N. The U-valley minima are assumed at the midpoint between the M and L symmetry points, with six equivalent valleys. The extracted band parameters for the six binary and ternary nitride alloys are given in Table 4.

**Table 4:** Material parameters used for mole fraction x = 0.2 in our Monte Carlo simulation.

Parameters	GaN	AlN	InN	Al_0.2_Ga_0.8_N	In_0.2_Ga_0.8_N	In_0.2_Al_0.8_N
M (kg/m^3^)	6087	3230	6240	5515.6	6117.6	3832
v_s_ (m/s)	7619	9060	3780	7907	6851.2	8004
D_a_ (eV)	8.3	6.2	4.76	7.88	7.05	5.37
${\varepsilon }_{s}$	9.7	8.5	15.3	9.46	10.82	9.86
${\varepsilon }_{\infty }$	5.28	4.77	8.4	5.18	5.904	5.496
${m}_{\Gamma }$	0.2	0.48	0.04	0.256	0.168	0.392
${m}_{\mathrm{U}}$	0.4	1	0.25	0.52	0.53	0.85
${\mathrm{m}}_{{\Gamma }_{3}}$	0.6	1	1	0.68	0.52	1
$U-\Gamma$(eV)	1.34	0.7	2.71	1.21	1.614	1.01
${\Gamma }_{3}-\Gamma$(eV)	2.14	1.0	1.78	1.91	2.068	1.156
Equivalent valleys						
$\Gamma$	1					
U	6					
${\Gamma }_{3}$	1					
${\hslash \omega }_{LO}$(meV)	0.091	0.099	0.073	0.093	0.087	0.094
${D}_{ij}$(eV m^-1^)	10^11^					
${\hslash \omega }_{ij}$(meV)	0.073	0.099	0.029	0.078	0.06	0.085
$\alpha$(eV^-1^) Nonparaboli-city						
$\Gamma$	0.19	0.04	1.32	1.16	0.42	0.299
U	0.03	0	0.23	0.06	0.07	0.046
${\Gamma }_{3}$	0.08	0	0	0.023	0.061	0.0
${P}_{z}$(C/m^2^)	0.38	0.92	0.38	0.48	0.38	0.811

M: mass density ${v}_{s}$: Sound velocity, ${D}_{a}$: acoustic deformation potential, ɛ_s_: static dielectric constant, ${\varepsilon }_{\infty }$: High Frequency dielectric constant, ${D}_{ij}$: Intervalley Deformation Potentials, $\alpha$: non-parabolicity factor and ${P}_{z}$: piezoelectric constant are taken from reference^10,11^.

m_ɼ_: Effective mass at Γ valley, m_U_: Effective mass at U valley, m_ɼ3_: Effective mass at ${\Gamma }_{3}$ valley, $U-\Gamma , {\Gamma }_{3}-\Gamma$ are valley separation, ${\hslash \omega }_{LO}$: Optical Phonon energies, ${\hslash \omega }_{ij}$: Intervalley Phonon Energies are extracted from the E–k data obtained through FullBand simulator.

Such structures have been obtained for all the other alloys considered here. The accuracy of simulated band gap results obtained from FullBand simulator is tested against experimental results and also compared against results obtained with various DFT based approaches. Our extracted bandgap results show excellent agreement with the experimental results as compared to the DFT-LDA, DFT-PBE and DFT-HSE results which are clearly reflected from Table 3. The outcome of electron transport on three valleys over electronic band structure using Monte Carlo technique has been tabulated for binary and ternary group-III nitrides, however for brevity we have shown only few graphical results i.e. at ambient temperature 300 K and doping dose 10^22^ m^−3^. Further, on the basis of results, a generalized innovative mobility model formulation is underway for all binary and ternary III–V nitride alloys.

**Figure 2:**
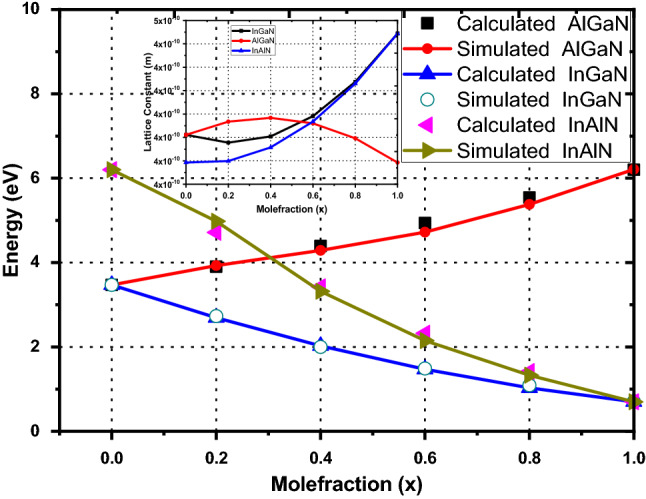
Energy gap as a function of mole fraction *x* for ternary alloys and comparison with calculated results based on Vegard’s law using reported bowing parameters^11^. Satellite Figure represents variation of lattice constant of ternary alloys with mole fraction ‘x’.

The comparison of the variation of simulated-energy gaps with previously reported values of energy gaps calculated using modified Vegard’s law^19–22^, as a function of mole fraction x for ternary nitride alloys is also shown in Table 3 and Fig. 2. Our simulated-energy gap as a function of mole fraction exhibits good agreement, with the results reported in literature^19–22^. Different values of the bowing parameter for a particular ternary nitride alloy are reported in literature for fitting the energy gap values^5,31^. In current analysis, alloy disorder effect along with, Table 2, under virtual crystal approximation is used to interpolate the lattice constant binary alloys to obtain the lattice constant of ternary alloys which subsequently leads to determination of energy gaps. The increasing degree of disorder causing the translational symmetry breaking and pronounced carrier localization, is included in the determined lattice parameter of ternary alloy and used in the FullBand simulator to determine the energy band gaps^32^. Variation of lattice parameter of the ternary nitride alloys with mole fraction ‘x’ is of parabolic nature as shown in the inset of Fig. 2. In_0.2_Ga_0.8_N and In_0.2_Al_0.8_N show small variation in lattice constant up to 4% Indium content beyond which the lattice constant increases sharply. For Al_0.2_Ga_0.8_N the behavior is inverse to that exhibited by the In-based ternary alloys. The optimum alloy disorder parameters used in present analysis are listed in Table 2. The extracted band parameters—ε_s_ ε_∞_ m_Г_ m_U_ m_Г3_, U-Г, Г_3_-Г, Г, U, Г_3_, ħω_LO_ , ħω_ij_ and α—from full band structure of each binary and ternary nitride alloys, are given in Table 4. However, the full band structure of Al_0.2_Ga_0.8_N only is shown here.

**Figure 3:**
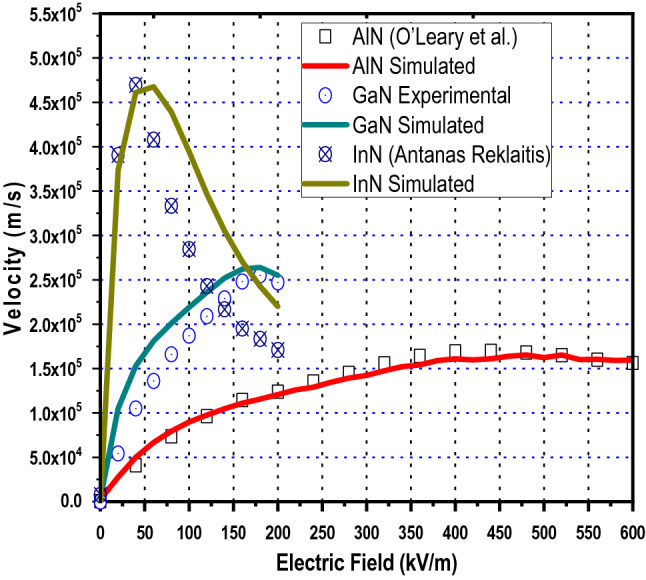
The Comparison of simulated electron drift velocity as a function of applied electric field with the reported literature. Data from present work is displayed as solid lines where as experimental/theoretical results are depicted by open circles, squares and triangles for AlN^33^, GaN^1^ and InN^34^ respectively.

The computation of electron drift velocity at different temperatures and various doping densities have been carried out on three valleys of full band structure. However data at room temperature only is given in Table 5. The peak values of drift velocity can be used for proposing generalized carrier mobility model for binary and ternary nitride alloys. The accuracy of the mobility model proposed in this paper has been verified against both the theoretical and experimental results reported in the literature. The model verification for n-type GaN^1^, AlN^33^ and InN^34^ is performed at various strengths of applied electric field, Fig. 3. The drift velocities simulated for binary nitride alloys have been compared with experimental/theoretical values reported in literature and the comparison is shown in Fig. 3. Notably, a close agreement has been found between our results and those reported in literature. Here, the highest peak-mobility 4.68 × 10^5^ m^2^/kV -s, is obtained in InN material, at 60 kV/m electric field, Fig. 3, beyond which there is a noticeable difference between our simulated-drift-velocity and that reported by Antanas Reklaitis^34^ . Our reported drift-velocity values are higher and the difference is due to selection of different type of scattering phenomena—acoustic, intervalley, optical (Polar), piezoelectric, ionized impurity, Coulomb and surface roughness-as well as due to selection of the optimum band parameter-values i.e. mass density M, sound velocity ${\mathrm{v}}_{\mathrm{s}}$, acoustic deformation potential ${D}_{a}$, intervalley deformation potentials ${D}_{ij}$, non-parabolicity factor $\alpha$, piezoelectric constant ${P}_{z}$ (Table 4) and the rest parameters extracted from the full band diagram shown in Fig. 1. The simulated-peak-velocity for GaN is quite close to the experimental velocity reported by Jonathan Marini et al.^1^, Fig. 3. Further, the drift velocity simulated by our model for AlN alloy matches exactly with the results reported by S. K. O’Leary et al.^33^*.* as shown in Fig. 3. Thus our present mobility model justifies the results previously reported in literature. The minor differences observed in the present simulation velocity-curve and already reported experimental velocity-curves reproduced here for GaN and InN is attributed to valley separation parameters which are responsible for the intervalley electrons transfer. Our valley separation parameters have been extracted from full energy band diagram obtained using FullBand simulator. Also the electron effective mass plays significant role in deciding the intervalley transitions in terms of valley occupation factor. Thus the analysis of electron transport done here on three valleys over full band structure extracts the accurate band parameters and hence the present model is suitable for actual prediction of transportation of electrons on the band structure under external applied electric field as is seen in Fig. 3.

**Table 5:** The comparison of ‘peak electron drift velocities v_d(sat)_ occurring at certain applied electric field.

Material	Without P_s_		With Piezoelectric Scattering (P_s_)					
	N_d_ = 10^22^ m^−3^		N_d_ = 10^21^ m^−3^		N_d_ = 10^22^ m^−3^		N_d_ = 10^23^ m^−3^	
	v_d(sat)_	$\varepsilon$	v_d(sat)_	$\varepsilon$	v_d(sat)_	$\varepsilon$	v_d(sat)_	$\varepsilon$
GaN	338,241.76	240	332,702.11	260	336,347.62	240	335,330.14	240
AlN	175,340.32	420	165,126.54	480	165,357.21	480	164,567.43	500
InN	495,755.11	40	468,867.88	60	468,405.97	60	467,559.28	60
Al_0.2_Ga_0.8_N	305,409.52	300	300,118.99	320	300,042.37	320	300,383.95	320
Al_0.4_Ga_0.6_N	282,982.04	360	277,402.67	360	276,870.74	360	276,513.34	380
In_0.2_Ga_0.8_N	328,157.22	220	323,701.12	220	323,543.69	220	322,908.79	240
In_0.4_Ga_0.6_N	332,536.42	180	326,761.45	180	326,562.54	200	325,739.35	200
In_0.2_Al_0.8_N	228,558.30	540	219,712.81	580	219,936.09	580	217,867.01	540
In_0.4_Al_0.6_N	230,890.69	440	221,966.17	480	221,816.73	480	221,207.67	460

$\varepsilon$’ for various doping densities at room temperature 300 K under piezoelectric scattering mechanism off and on. The ratio of v_d(sat)_ to $\varepsilon$ gives the mobility.

**Figure 4:**
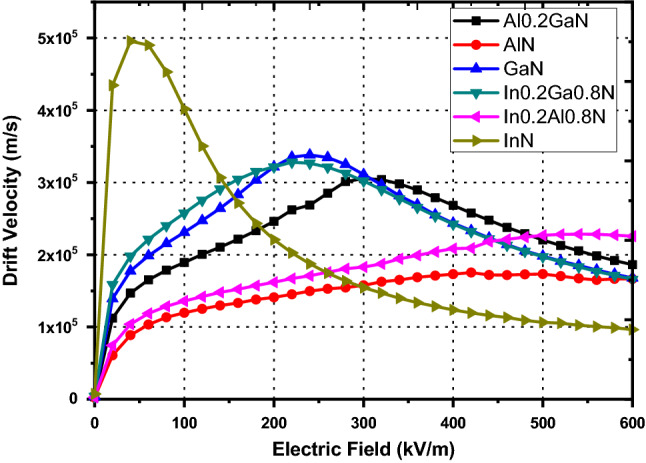
Carrier drift velocity with respect to the electric field (kV/m) of binary and ternary alloys without piezoelectric Scattering and doping density 10^22^ m^−3^ at 300 K.

**Figure 5:**
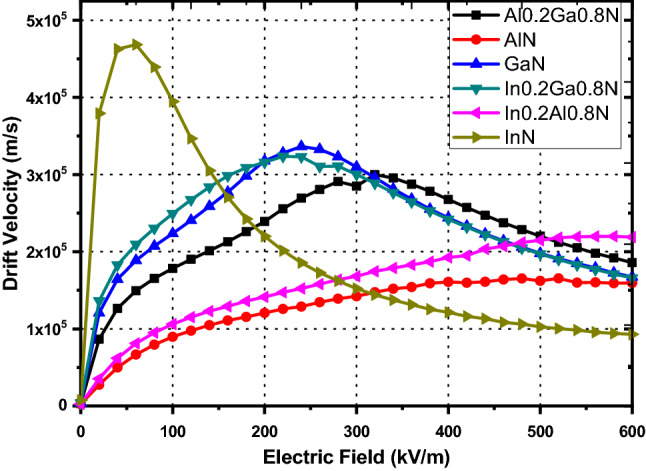
Carrier drift velocity using the model presented here, with respect to the electric field (kV/m) of binary and ternary alloys with piezoelectric Scattering and doping density 10^22^ m^−3^ at 300 K.

Therefore, using this model electron drift velocity at room temperature as a function of electric field for GaN, AlN, InN, Al_0.2_Ga_0.8_N, In_0.2_Ga_0.8_N, and In_0.2_Al_0.8_N is displayed in Figs. 4 and 5. The simulated-velocity curves shown in Fig. 3 for model calibration purpose are included in Fig. 5. The drift velocity associated with different nitride alloys is simulated by considering the maximum piezoelectric scattering rate at varying doping densities and different operating temperatures. The simulation data for peak-drift velocity along with the corresponding applied electric field strength ε under various operating conditions are tabulated in Table 5. The effect of piezoelectric scattering on drift velocity has been analyzed by removing the terms representing piezoelectric scattering from the present model. The drift velocity values in the absence and in the presence of piezoelectric scattering are illustrated in Figs. 4 and 5 respectively. With a doping density of 10^22^ m^−3^ and absence of piezoelectric scattering, the peak-drift-velocity values come out to be 338,241.76, 175,340.32, 495,755.11, 305,409.52, 282,982.04, 328,157.22, 332,536.42, 228,558.30 and 230,890.69 m/s for GaN, AlN, InN, Al_0.2_Ga_0.8_N, Al_0.4_Ga_0.6_N, In_0.2_Ga_0.8_N, In_0.4_Ga_0.6_N, In_0.2_Al_0.8_N and In_0.4_Al_0.6_N respectively, which are comparatively higher than the corresponding values in presence of piezoelectric scattering, as expected. Also the peak-drift-velocities occur at comparatively lower applied field strengths, Table 5. This prominent difference, due to absence of piezoelectric scattering mechanism, is observed in all nitride alloys. As can also be seen from Fig. 4 the peak-drift-velocity increases in magnitude and shifts to left side i.e. towards lower applied electric field, when piezoelectric scattering is absent over the three valleys. Maximum variation in drift velocity is found in InN and its composite alloys, due to high piezoelectric scattering rate, which is also evident from Table 5. When piezoelectric scattering term is taken into account and the doping density is taken as 10^22^ m^−3^, again the highest peak-drift-velocity is exhibited by InN and at a much lower electric field as compared to other nitride alloys, Fig. 5. This study proves that piezoelectric scattering effect can provide useful details of physical effects inside responsible for degradation of electron mobility and could be minimized through application of reverse piezoelectric effect in the films through application of appropriate electric field. A close look at Figs. 4 and 5 clearly demonstrates the fact for InN and GaN. The analysis proposed by Jin Zhang^35^ also confirms that under the influence of external electric field the Young’s modulus of GaN monolayer changes, thus changing the phonon group velocity. It was found that the applied field induces in-plane stress in the monolayer GaN due to the inverse piezoelectric effect. It changes the lattice anharmonicity as well as affects the phonon mean free path. The in-plane stress may generate the buckling instability in long GaN monolayers and significantly reduce the phonon mean free path.

**Figure 6:**
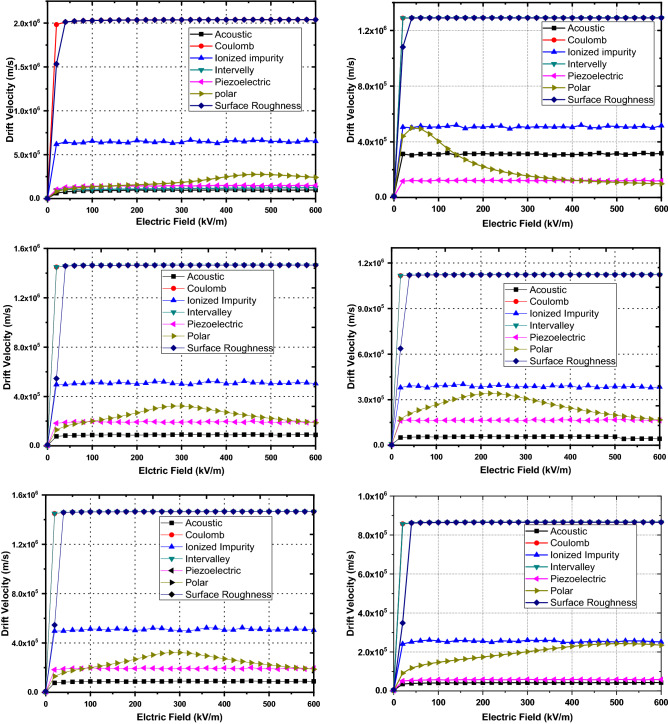
(**a**) AlN alloy Carrier drift velocity with respect to electric field (E) with separate scattering mechanisms and doping density 1022 m^−3^ at 300 K. (**b**) GaN alloy Carrier drift velocity with respect to electric field (E) with separate scattering mechanisms and doping density 1022 m^−3^ at 300 K. (**c**) InN alloy Carrier drift velocity with respect to electric field (E) with separate scattering mechanisms and doping density 1022 at 300 K. (**d**) Al0.2Ga0.8N alloy Carrier drift velocity with respect to Electric Field (E) with separate scattering mechanisms and doping density 1022 m^−3^ at 300 K. (**e**) In0.2Ga0.8N alloy Carrier drift velocity with respect to Electric Field (E) with separate scattering mechanisms and doping density 1022 m^−3^ at 300 K. (**f**) In0.2Al0.8N alloy Carrier drift velocity with respect to Electric Field (E) with separate scattering mechanisms and doping density 1022 m^−3^ at 300 K.

The effect of separate scattering mechanisms on the carrier drift velocity with respect to applied electric field is shown in Fig. 6 a–f for doping density 10^22^ m^−3^ at 300 K for binary and ternary group-III nitrides. The initial variation of electron drift velocity for each scattering mechanism shows linear dependence on applied electric field followed by state of saturation resulting in constant drift velocity for increasing electric field. The saturated drift velocity justifies the maximum valley occupation of electrons and intervalley transfer of electrons. All the scattering mechanisms show similar trend except polar optical scattering which depicts the nonlinear behavior. The nonlinear behavior can be explained through change in energy and mass of carrier after polar optical scattering event take place. At a given temperature T, the polar optical scattering will be as large as $\mathrm{\hslash }{\omega }_{\mathrm{LO}}$ and is close to K_B_T. On the other hand, the polar optical scattering dependence on the square root of the effective mass is easy to understand^2^, since the interaction with the long-range field associated to longitudinal phonon modes must grow with the electron localization, and thus with the mass. When $\mathrm{E}<\hslash {\omega }_{\mathrm{LO}}$, only absorption of $\mathrm{\hslash }{\omega }_{\mathrm{LO}}$ phonons is possible; when E comes across $\mathrm{\hslash }{\omega }_{\mathrm{LO}}$, carriers can absorb and emit, thus polar optical scattering is suddenly suppressed; finally, when $\mathrm{E}\gg \mathrm{\hslash }{\omega }_{\mathrm{LO}}$ the scattering probability becomes more and more unlikely and polar optical scattering rate increases. It is clearly seen from Fig. 6 that polarization Coulomb field (PCF) scattering along with acoustic and intervalley scatterings is the dominant scattering mechanism and responsible for low electron mobility. The nonlinear behavior of polar optical scattering mechanism also tends to reduce the electron mobility. The effect of ionized impurity and surface roughness scatterings on the electron mobility is found to have negligible impact. The piezoelectric Coulomb field scattering has a stronger influence on the electron transport in all binary and ternary alloys.

**Figure 7:**
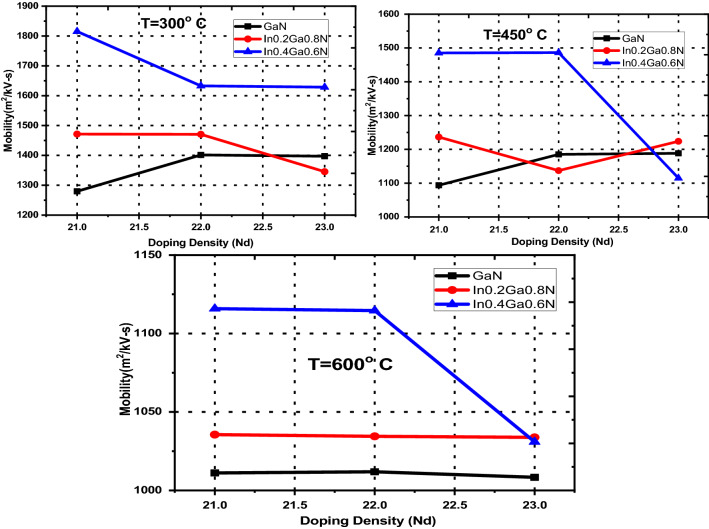
The variation of carrier mobility in GaN and In_x_Ga_1−x_N (for x = 0.2 and x = 0.4) with variation of doping density N_d_ (m^−3^) at temperatures 300, 450 and 600 K using Table 6.

The relation between electron mobility, the ratio of drift velocity to applied electric field, and doping density at different temperatures (300, 450 and 600 K) shows nonlinear variation due to dominant piezoelectric Coulomb field scattering in the lowest valley, Г, along with different behavior for the various nitride alloys studied here, Fig. 7. This electron mobility is also dependent on the energy separation between lowest and other secondary valleys i.e. U, Г_3_, as depicted in Table 6.

**Table 6:** The mobility data of nitride alloys with variation of doping density at different temperatures.

Materials	N_d_	${\mu }_{300}$	${\mu }_{450}$	${\mu }_{600}$
GaN	10^21^	1279.62	1093.64	1011.16
	10^22^	1401.45	1185.04	1011.88
	10^23^	1397.21	1188.30	1008.36
AlN	10^21^	344.01	262.24	223.01
	10^22^	344.49	282.75	225.32
	10^23^	329.13	269.66	239.40
InN	10^21^	7814.46	6952.23	6123.57
	10^22^	7806.77	6960.48	6115.73
	10^23^	7792.65	6941.83	6096.48
Al_0.2_Ga_0.8_N	10^21^	937.87	815.39	705.12
	10^22^	937.63	864.19	705.67
	10^23^	938.70	812.89	702.72
Al_0.4_Ga_0.6_N	10^21^	770.56	639.59	530.68
	10^22^	769.08	602.41	556.00
	10^23^	727.67	638.49	529.72
In_0.2_Ga_0.8_N	10^21^	1471.37	1236.37	1035.57
	10^22^	1470.65	1136.97	1034.46
	10^23^	1345.45	1223.68	1033.84
In_0.4_Ga_0.6_N	10^21^	1815.34	1485.20	1115.84
	10^22^	1632.81	1486.31	1114.59
	10^23^	1628.69	1114.80	1030.95
In_0.2_Al_0.8_N	10^21^	378.81	335.17	308.89
	10^22^	379.20	345.14	300.30
	10^23^	403.60	346.10	300.28
In_0.4_Al_0.6_N	10^21^	439.44	403.11	313.53
	10^22^	462.12	403.32	337.07
	10^23^	480.89	402.52	313.33

The ${\mu }_{T}$ (m^2^/kV-s) is electron mobility with suffix representing operating temperature in Kelvin,N_d_ is doping density (m^−3^) and piezoelectric scattering is taken into account.

**Figure 8:**
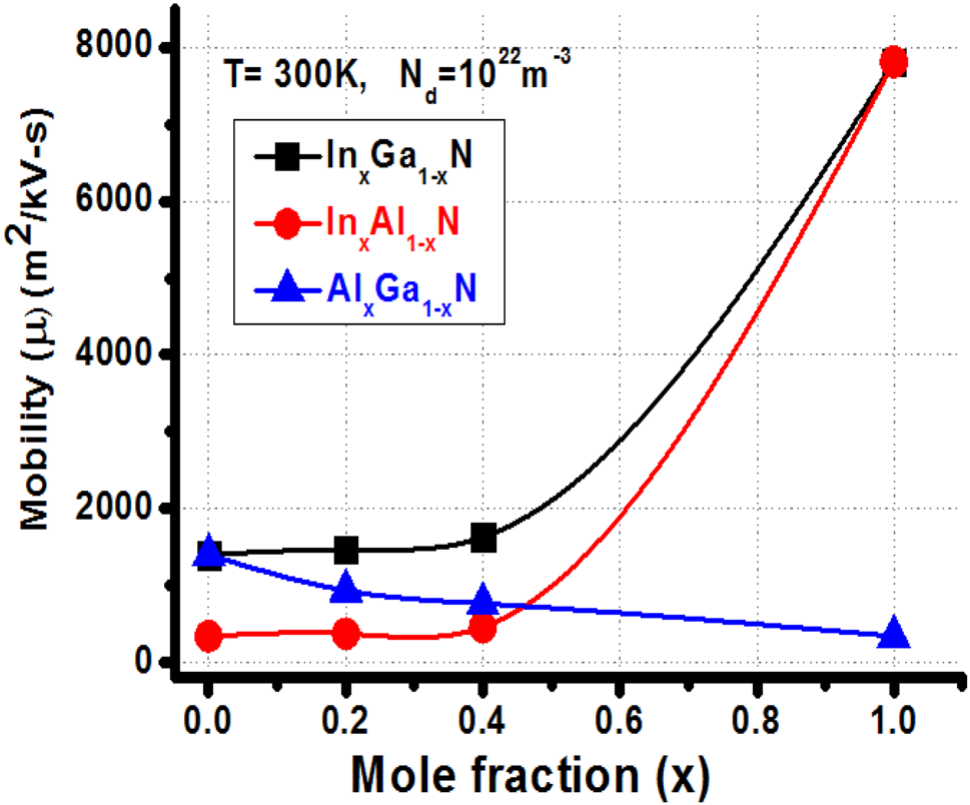
The carrier mobility in nitride alloys with variation of mole fraction x in ternary alloys at 300 K for doping density 10^22^ m^−3^ using Table 6. T is the operating temperature and N_d_ (m^−3^) is doping density.

Regarding mobility in ternary nitride alloys the mole fraction x also plays important role. On changing x, from 0 to 1 i.e. at extreme values of x, ideally the mobility should follow the behavior of binary alloys but the results obtained here are in contradiction indicating a nonlinear response. The nonlinear dependence is reflected in Fig. 8 as with increasing indium content in In_x_Ga_1−x_N and In_x_Al_1−x_N the mobility increases whereas for increasing aluminium content in Al_x_Ga_1−x_N the mobility decreases. The behavior in mobility shown by Al_x_Ga_1−x_N in Fig. 8 is described in terms of relation between energy and mole fraction as depicted in Fig. 3. Increasing the Al content in Al_x_Ga_1−x_N, the energy increases, while increasing In content in In_x_Ga_1−x_N and In_x_Al_1−x_N, the energy decreases. The probability of scattering events increases with increase in energy values in turn degrading the mobility of carriers. The data for Fig. 8 is taken from Table 6 for 300 K and doping density 10^22^ m^−3^. It should be noted that the energy separation varies with variation in x. The electrons mobility data for other doping densities, shown in Table 6 also exhibit nonlinear behavior.

In GaN alloy, carrier mobility is higher when piezoelectric scattering mechanism is absent. At low doping dose 10^21^ m^−3^ mobility is less, increases at moderate dose 10^22^ m^−3^ and again decreases for higher dose 10^23^ m^−3^, Table 6. In fact the binary alloy GaN’s mobility trend with varying doping density should be followed by its ternary alloys, but the mobility rule observed with GaN is not found true for In_x_Ga_1−x_N or Al_x_Ga_1−x_N. The reason is defined with the difference in energy separation values and nonlinearity effect in lowest valley. The varying effective masses of electrons in different valleys are also a deciding factor for determination of the nonlinear electron drift velocity and hence mobility in ternary nitride alloys. The other two ternary alloys with different mole fractions do not follow the similar mobility rule endorsing the nonlinear effect. At room temperature with varying doping dose, Al_0.4_Ga_0.6_N shows continuous decrease in peak electron drift velocity observed at higher electric field; however in case of Al_0.2_Ga_0.8_N the mobility first decreases and then increases though the variation is quite small. Moreover the peak electron drift velocity is observed at comparatively lower electric fields.

The effects of temperature on the carrier mobility for nitride alloys are estimated rigorously with the variation in doping dose and mole fraction of the ternary alloys. The electron mobility data for different alloys with various doping densities and different operating temperatures, in Kelvin, are given in Table 6, Fig. 8. Most of the nitride alloys show decrease in mobility values with increase in operating temperature and doping density, except GaN. Its composites justify non-linear mobility effects with doping and temperature variations. The effect can be explained on the basis of energy separation and carrier dynamics over three valleys. At moderate doping (10^22^ m^−3^) and high temperature (450 K), the electrons scattered to U-valley and due to higher effective mass, remains in same valley. The higher mobility (= 7814.46 m^2^/kV- s) is observed for InN alloy under low doping dose (10^21^ m^−3^) operating at room temperature (300 K). Increased doping dose and higher operating temperature initiate the higher scattering probabilities with higher electron energy and hence reduces the mobility.

## Conclusions

Optimized Empirical Pseudopotential Method (EPM) in conjunction with Virtual Crystal Approximation (VCA) and the compositional disorder effect inbuilt in TNL’s FullBand simulator is used here to extract the electronic materials parameters of wurtzite nitride alloys. The band gap results ensure excellent agreement with the experiments and show superior results over other various DFT techniques based theoretical approaches. Another beauty of this in-house developed model from FullBand simulator is that it requires only lattice constant, as input, of associated binary alloys and interpolated lattice constant of ternary nitride alloys to accurately predict the full band structure. Good agreement have been found between simulated results and experimental results reported by others regarding electron mobility of binary nitride materials GaN, InN and AlN which is attributed to using optimized band structures and estimation of electron mobility based on different scattering mechanisms. Also the energy separation values vary with variation in x of ternary nitride alloys. The maximum influence of PCF scattering on binary and ternary nitride alloys are observed during electrons transport under applied electric field and is found to be responsible for mobility reduction. The nonlinear mobility behavior is noticed for GaN and its alloys. The mobility results obtained in present work indicate that electron mobility can be increased by reducing the PCF (piezoelectric and Coulomb field) scattering mechanisms. The relation between electron mobility and doping density at different temperatures, 300, 450 and 600 K, shows nonlinear variation in the lowest valley, Г, along with different behavior for the various nitride alloys studied here. Mobility in ternary nitride alloys varies non-linearly with the mole fraction x. For example, at temperature 300 K and doping density 10^22^ m^−3^, increasing indium content in In_x_Ga_1−x_N and In_x_Al_1−x_N increases the mobility whereas increasing aluminium content in Al_x_Ga_1−x_N decreases the mobility. The electrons mobility data for other doping densities, considered here also exhibit nonlinear behavior. The electron mobility simulated results of each binary nitride material have been compared with experimental results under appropriate conditions and good agreement has been found between them.
